# Supplementary material for: Enhancing quantum teleportation efficacy with noiseless linear amplification
Source: Nat Commun. 2023 Aug 7;14:4745. doi: 10.1038/s41467-023-40438-z (PMC10406873; doi:10.1038/s41467-023-40438-z)
Supplement: Supplementary file 1 — Supplementary Information [file 41467_2023_40438_MOESM1_ESM.pdf]

Enhancing quantum teleportation efficacy with noiseless amplification: supplementary note

Jie Zhao,[1,2] Hao Jeng,[1] Lorcán O. Conlon,[1] Spyros Tserkis,[1] Biveen Shajilal,[1]
Kui Liu,[3] Timothy C. Ralph,[4] Syed M Assad,[1] and Ping Koy Lam[1,5*]
[1]*Centre for Quantum Computation and Communication Technology, Department of Quantum Science and Technology, Research School of Physics, The Australian National University, Canberra ACT 2601, Australia.*
[2]*Joint Quantum Institute, National Institute of Standard and Technology and University of Maryland, College park, 20742, Maryland, USA.*
[3]*State key laboratory of quantum optics and quantum optics devices, Institute of Opto-Electronics, Collaborative Innovation Center of Extreme Optics, Shanxi University, Taiyuan 030006, China.*
[4]*Centre for Quantum Computation and Communication Technology, School of Mathematics and Physics, University of Queensland, St. Lucia, QLD 4072, Australia.*
[5]*Institute of Materials Research and Engineering, Agency for Science Technology and Research (A*STAR), 2 Fusionopolis Way, Innovis 138634, Singapore.*
* email: ping.lam@anu.edu.au

(Dated: August 4, 2023)

I. SUPPLEMENTARY NOTE 1: THEORETICAL MODEL

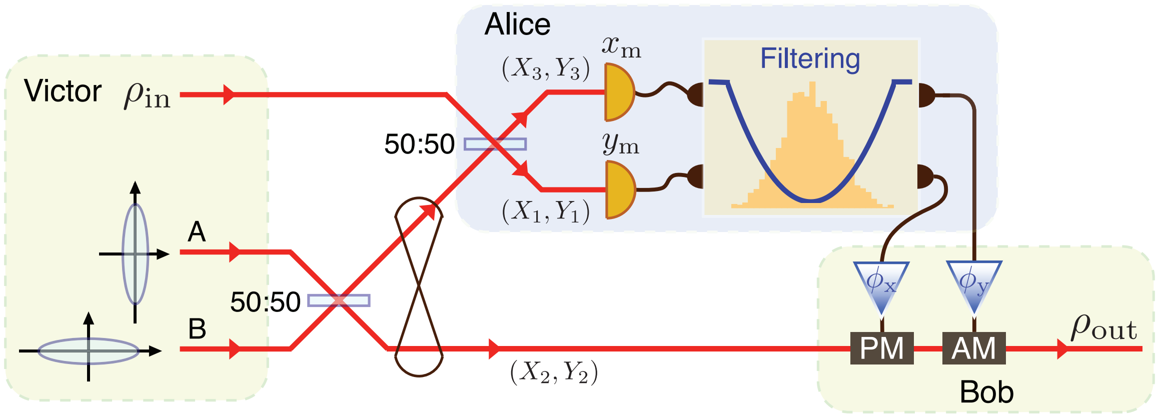

Supplementary Figure 1: Schematic drawing of our heralded quantum teleporter. The notations X_n and Y_n refer to the amplitude and phase quadrature operators of mode n, respectively.

Here we provide the theoretical modelling of our teleporter by investigating the evolution of the system's quadrature field operators. The input-output relations for the field operators are derived in the following. The schematic of our teleporter is illustrated in Supplementary Figure 1.

We start with combining two single-mode squeezed states A and B on a 50:50 beamsplitter. This creates a two-mode squeezed state, also known as the Einstein-Podolski-Rosen (EPR) state. The covariance matrix of the EPR state is given by

$$\mathbf{C} = \begin{pmatrix} C_{11} & 0 & C_{13} & 0 \\ 0 & C_{22} & 0 & C_{24} \\ C_{13} & 0 & C_{11} & 0 \\ 0 & C_{24} & 0 & C_{22} \end{pmatrix}. \tag{1}$$

The matrix elements are given by

$$\begin{aligned} C_{11} &= \frac{e^{-2r_{\mathrm{Ax}}} + e^{2r_{\mathrm{Bx}}}}{2}, \quad C_{22} = \frac{e^{-2r_{\mathrm{By}}} + e^{2r_{\mathrm{Ay}}}}{2}, \\ C_{13} &= \frac{e^{2r_{\mathrm{Bx}}} - e^{-2r_{\mathrm{Ax}}}}{2}, \quad C_{24} = \frac{e^{-2r_{\mathrm{By}}} - e^{2r_{\mathrm{Ay}}}}{2}. \end{aligned} \tag{2}$$

Here $r_{\mathrm{Ax(y)}}$ and $r_{\mathrm{Bx(y)}}$ refer to the squeezing parameters of the initial single-mode squeezed states A and B, respectively. The subscript x(y) denotes the amplitude (phase) quadrature. Note that the diagonal elements C_{11} and C_{22} represent the thermal noise variance in the quadratures of the EPR state, while the off-diagonal elements C_{13} and C_{24} are the EPR correlations between the quadratures. In circumstances where the initial squeezed sources are pure and identical, i.e. $r_{\mathrm{Ax}} = r_{\mathrm{Ay}} = r_{\mathrm{Bx}} = r_{\mathrm{By}} = r$, the covariance matrix in Supplementary Equation (1) specializes to its more commonly used form,

$$\mathbf{C}_{\mathrm{EPR}} = \begin{pmatrix} \cosh r\mathbf{I} & \sinh r\mathbf{Z} \\ \sinh r\mathbf{Z} & \cosh r\mathbf{I} \end{pmatrix}, \tag{3}$$

where $\mathbf{I}$ is the identity matrix and $\mathbf{Z} := \mathrm{diag}(1, -1)$. An unknown input state is then inserted into our device and coupled with one sub-mode of the EPR state on a beamsplitter with transmittance of 50%, giving rise to the combined state with covariance matrix $\mathbf{C}$ that takes the form of

$$\mathbf{C}' = \begin{pmatrix} C_{11} & 0 & C_{13}/\sqrt{2} & 0 & -C_{13}/\sqrt{2} & 0 \\ 0 & C_{22} & 0 & C_{24}/\sqrt{2} & 0 & -C_{24}/\sqrt{2} \\ C_{13}/\sqrt{2} & 0 & (\langle(\Delta X_{\mathrm{in}})^2\rangle + C_{11})/2 & 0 & (\langle(\Delta X_{\mathrm{in}})^2\rangle - C_{11})/2 & 0 \\ 0 & C_{24}/\sqrt{2} & 0 & (C_{22} + \langle(\Delta Y_{\mathrm{in}})^2\rangle)/2 & 0 & (\langle(\Delta Y_{\mathrm{in}})^2\rangle - C_{22})/2 \\ -C_{13}/\sqrt{2} & 0 & (\langle(\Delta X_{\mathrm{in}})^2\rangle - C_{11})/2 & 0 & (C_{11} + \langle(\Delta X_{\mathrm{in}})^2\rangle)/2 & 0 \\ 0 & -C_{24}/\sqrt{2} & 0 & (\langle(\Delta Y_{\mathrm{in}})^2\rangle - C_{22})/2 & 0 & (C_{22} + \langle(\Delta Y_{\mathrm{in}})^2\rangle)/2 \end{pmatrix}. \tag{4}$$

Here $\langle(\Delta X_{\mathrm{in}}(Y_{\mathrm{in}}))^2\rangle$ is the noise variance in the amplitude (phase) quadrature of the input state. The corresponding quadrature field operators are defined as

$$\mathbf{X} = (X_2, Y_2, X_1, Y_1, X_3, Y_3)^{\mathrm{T}}, \tag{5}$$

where the subscripts $1, 2, 3$ denote the respective spatial modes as illustrated in Supplementary Figure 1. Elements of the covariance matrix $\mathbf{C}'$ in Supplementary Equation (4) are defined accordingly as $\mathbf{C}'_{ij} := \frac{1}{2}\langle\{\Delta\mathbf{X}_i, \Delta\mathbf{X}_j\}\rangle$, where $\Delta\mathbf{X}_i := \mathbf{X}_i - \langle\mathbf{X}_i\rangle$ and $\{,\}$ is the anticommutator. As an example, $C_{11}=\mathbf{C}'_{11}$ equals to $\frac{1}{2}\langle\{\Delta X_2, \Delta X_2\}\rangle$, that is the noise variance in amplitude of mode 2. Therefore, the covariance between the amplitude quadratures of modes n and m can be written as $\langle\Delta X_n \Delta X_m\rangle$. For brevity, Δ is omitted in the following derivations. The two outgoing modes of the beamsplitter are subject to a dual-quadrature homodyne performed by Alice to measure the amplitude and phase quadratures simultaneously. The EPR sub-mode on Bob's side evolves accordingly conditioned upon Alice's measurement outcome $\alpha_m = (x_m + iy_m)/\sqrt{2}$. It follows that the conditional amplitude mean and variance of the transmitted EPR mode take the form

$$\begin{aligned} \mu_{X_2|\bar{x}_m} &= \langle X_2\rangle + \frac{\langle X_2X_3\rangle}{\langle X_3X_3\rangle}(\bar{x}_m - \langle X_3\rangle) = \frac{-\sqrt{2}C_{13}}{\langle X_{\mathrm{in}}X_{\mathrm{in}}\rangle + C_{11}}\left(\bar{x}_m - \frac{\langle X_{\mathrm{in}}\rangle}{\sqrt{2}}\right)^T, \\ \Sigma_{X_2|\bar{x}_m} &= \langle X_2X_2\rangle - \frac{\langle X_2X_3\rangle\langle X_3X_2\rangle}{\langle X_3X_3\rangle} = C_{11} - \frac{C_{13}^2}{\langle X_{\mathrm{in}}X_{\mathrm{in}}\rangle + C_{11}}. \end{aligned} \tag{6}$$

To obtain the average output mean $\mu_{X_2|\bar{x}_m}$, one needs to integrate over all possible outcomes, denoted as $\bar{x}_m$. The conditional variance $\Sigma_{X_2|\bar{x}_m}$ refers to the variance of the amplitude quadrature of mode 2 conditioned upon the measurement outcomes of X_3. It quantifies how well one can estimate the amplitude quadrature of mode 2 based on observed amplitude values of mode 3 up to, on average, some noise $\langle X_3X_3\rangle$. For example, given an EPR state with its two constituents being modes a and b the conditional variance would be [1]

$$\Sigma_{X_{\mathrm{a}}|\bar{x}_{\mathrm{b}}} = \langle X_{\mathrm{a}}X_{\mathrm{a}}\rangle\left(1 - \frac{\langle X_{\mathrm{a}}X_{\mathrm{b}}\rangle^2}{\langle X_{\mathrm{a}}X_{\mathrm{a}}\rangle\langle X_{\mathrm{b}}X_{\mathrm{b}}\rangle}\right) = \frac{1}{\cosh(2r)}. \tag{7}$$

Estimation of the quadrature amplitudes of one sub-mode based on measurement outcomes of the other is subject to the maximum amount of noise when $r = 0$; otherwise, perfect EPR correlation is attainable when $r \to \infty$, leading to $\Sigma \to 0$. Perfect estimation is therefore achievable.

Similarly, one can obtain the conditional mean and variance for the phase quadrature

$$\begin{aligned}\mu_{Y_2|\bar{y}_m} &= \langle Y_2\rangle + \frac{\langle Y_2Y_1\rangle}{\langle Y_1Y_1\rangle}\left(\bar{y}_m - \langle Y_1\rangle\right) = \frac{\sqrt{2}C_{24}}{\langle Y_{\rm in}Y_{\rm in}\rangle + C_{22}}\left(\bar{y}_m - \frac{\langle Y_{\rm in}\rangle}{\sqrt{2}}\right)^T, \\ \Sigma_{Y_2|\bar{y}_m} &= \langle Y_2Y_2\rangle - \frac{\langle Y_2Y_1\rangle\langle Y_1Y_2\rangle}{\langle Y_1Y_1\rangle} = C_{22} - \frac{C_{24}^2}{\langle Y_{\rm in}Y_{\rm in}\rangle + C_{22}}.\end{aligned} \tag{8}$$

It is interesting to note that for coherent input states, the transmitted EPR sub-mode projects to a coherent state if a dual homodyne measurement is performed on the other sub-mode and the original squeezed states that comprise the EPR are identical (not necessarily pure), namely, $r_{\rm Ax(y)} = r_{\rm Bx(y)}$. Otherwise, when $r_{\rm Bx} > r_{\rm Ax}$–implying that the EPR ball in phase space is elongated along the amplitude quadrature–the conditional state is a phase-squeezed state, and vice versa. A distributed measurement-based squeezing gate can be constructed in this way, whereby optimum squeezing is obtained when Victor directly sends one of the initial squeezed states to Bob.

Unlike conventional quantum teleportation, the measurement outcomes are post selected with acceptance probability given by the following filter function [2]

$$f(\alpha_{\rm m}) \propto \exp\left[\left(|\alpha_{\rm m}|^2 - \alpha_{\rm c}^2\right)(1 - \frac{1}{g^2})\right]. \tag{9}$$

The post-selection, when followed by a rescaling that maps $\alpha_{\rm m}$ to $g\alpha$, effectively approximates a noiseless linear amplifier [2]. Here, g denotes the noiseless gain, while $\alpha_{\rm c}$ is the cut-off parameter that defines the operational regime of the amplification. In practice, all measurement outcomes with magnitude less than $\alpha_{\rm c}$, namely $|\alpha_m|<\alpha_{\rm c}$, are selected with probability specified in Supplementary Equation (9); outcomes that fall beyond the cut-off circle are kept with unity probability. On one hand, the inclusion of NLA effectively accomplishes the entanglement distillation: entanglement disguised under noise is distilled by increasing g and hence teleportation fidelity is enhanced, at the expense of a finite success probability; on the other hand, the post-selection avoids the noise penalty associated with Alice's dual homodyne measurement. By increasing g, the conjugate quadratures can be estimated with arbitrarily high accuracy, which is impossible by conventional means. By virtue of this purification process, perfect reconstruction of the initial input state can be achieved.

To be concrete, assume the measurement outcomes $\alpha_{\rm m}$ of the dual homodyne follow an unnormalized Gaussian distribution with mean α_0

$$p(\alpha_{\rm m}) \propto e^{-|\alpha_{\rm m}-\alpha_0|^2}. \tag{10}$$

The output distribution after post-selection becomes proportional to

$$p(\alpha_{\rm m})f(\alpha_{\rm m}) = e^{-|\alpha-\alpha_{\rm m}|^2}e^{\left(|\alpha|-\alpha_{\rm c}^2\right)\left(1-\frac{1}{g^2}\right)} = \frac{Ne^{(g^2-1)|\alpha_{\rm m}|^2}}{e^{\alpha_{\rm c}^2(1-1/g^2)}}e^{\frac{-|\alpha-g^2\alpha_{\rm m}|^2}{g^2}}, \tag{11}$$

where N is a normalization term. Overall, the post-selection gives rise to a displaced distribution with the input mean and variance both being amplified by g^2. Although this transformation is demonstrated for Gaussian input ensembles, it holds valid for other states due to its universality in emulating a physical noiseless linear amplifier [2, 3]. Upon post-selecting the dual-homodyne outcomes, the corresponding mode quadrature operators effectively undergo the following transformations

$$X_3 \to \tilde{X}_3, \quad Y_1 \to \tilde{Y}_1, \tag{12}$$

where

$$\langle\tilde{X}_3\rangle = g^2\langle X_3\rangle, \quad \langle\tilde{X}_3\tilde{X}_3\rangle = g^2\langle X_3X_3\rangle = g^2(\langle X_{\rm in}X_{\rm in}\rangle + C_{11})/2, \tag{13}$$

(14)

and

$$\langle\tilde{Y}_1\rangle = g^2\langle Y_1\rangle, \quad \langle\tilde{Y}_1\tilde{Y}_1\rangle = g^2\langle Y_1Y_1\rangle = g^2(\langle Y_{\rm in}Y_{\rm in}\rangle + C_{22})/2. \tag{15}$$

We emphasize that the transformation embodied in Supplementary Equation (12) does not represent a physical unitary process, but rather a change in the classical statistical properties of the modes' amplitude and phase quadratures.

For the transmitted EPR mode, post-selection should have no impact on its conditional statistical properties. To satisfy this requirement, the following relations must hold true

$$\frac{\langle\tilde{X}_2\tilde{X}_3\rangle}{\langle\tilde{X}_3\tilde{X}_3\rangle} = \frac{\langle X_2X_3\rangle}{\langle X_3X_3\rangle}, \quad \langle\tilde{X}_2\tilde{X}_2\rangle - \frac{\langle\tilde{X}_2\tilde{X}_3\rangle\langle\tilde{X}_3\tilde{X}_2\rangle}{\langle\tilde{X}_3\tilde{X}_3\rangle} = \langle X_2X_2\rangle - \frac{\langle X_2X_3\rangle\langle X_3X_2\rangle}{\langle X_3X_3\rangle}. \tag{16}$$

Similar relations can be derived for the phase quadrature. Elements of the covariance matrix after post-selection can be obtained using Supplementary Equations (16), (4), and (13):

$$\begin{aligned}
\langle \tilde{X}_2\tilde{X}_3\rangle &= \frac{\langle X_2X_3\rangle\langle \tilde{X}_3\tilde{X}_3\rangle}{\langle X_3X_3\rangle} = \frac{-C_{13}g^2}{\sqrt{2}}, \quad \langle \tilde{Y}_2\tilde{Y}_1\rangle = \frac{\langle Y_2Y_1\rangle\langle \tilde{Y}_1\tilde{Y}_1\rangle}{\langle Y_1Y_1\rangle} = \frac{C_{24}g^2}{\sqrt{2}},\\
\langle \tilde{X}_2\tilde{X}_2\rangle &= \Sigma_{X_2|\bar{x}_m} + \frac{\langle \tilde{X}_2\tilde{X}_3\rangle\langle \tilde{X}_3\tilde{X}_2\rangle}{\langle \tilde{X}_3\tilde{X}_3\rangle} = C_{11} + \frac{C_{13}^2(g^2-1)}{\langle X_{\rm in}X_{\rm in}\rangle + C_{11}},\\
\langle \tilde{Y}_2\tilde{Y}_2\rangle &= \Sigma_{Y_2|\bar{y}_m} + \frac{\langle \tilde{Y}_2\tilde{Y}_1\rangle\langle \tilde{Y}_1\tilde{Y}_2\rangle}{\langle \tilde{Y}_1\tilde{Y}_1\rangle} = C_{22} + \frac{C_{24}^2(g^2-1)}{\langle Y_{\rm in}Y_{\rm in}\rangle + C_{22}},
\end{aligned} \tag{17}$$

Following the same methodology, we now derive the conditional mean of the amplitude and phase quadratures of the EPR arm that is directly sent over to Bob. The mean values are conditioned upon the filtered dual-homodyne measurement outcomes at Alice's end:

$$\begin{aligned}
\tilde{\mu}_{X_2|\bar{\bar{x}}_m} &= \mu_{X_2|\bar{x}_m} + \frac{\langle \tilde{X}_2\tilde{X}_3\rangle}{\langle \tilde{X}_3\tilde{X}_3\rangle}\left(g^2\bar{x}_m - \frac{\langle X_{\rm in}\rangle}{\sqrt{2}}\right) = \frac{C_{13}\left(1-g^2\right)\langle X_{\rm in}\rangle}{\langle X_{\rm in}X_{\rm in}\rangle + C_{11}},\\
\tilde{\mu}_{Y_2|\bar{\bar{y}}_m} &= \mu_{Y_2|\bar{y}_m} + \frac{\langle \tilde{Y}_2\tilde{Y}_1\rangle}{\langle \tilde{Y}_1\tilde{Y}_1\rangle}(g^2\bar{y}_m - \frac{\langle Y_{\rm in}\rangle}{\sqrt{2}}) = \frac{C_{24}\left(g^2-1\right)\langle Y_{\rm in}\rangle}{\langle Y_{\rm in}Y_{\rm in}\rangle + C_{22}}.
\end{aligned} \tag{18}$$

The post-selected signals $\tilde{X}_3$ and $\tilde{Y}_1$ are rescaled electronically by $\phi_{\rm x(y)}$ prior to being sent to Bob through a classical channel. Bob performs a displacement operation onto his EPR mode using a pair of electro-optical modulators. Therefore, the mean quadrature amplitudes of the teleported state can be expressed as mean of the EPR mode 2 plus a displacement

$$\langle X_{\rm out}\rangle = \tilde{\mu}_{X_2|\bar{\bar{x}}_m} + \langle \tilde{X}_3\rangle = \tilde{\mu}_{X_2|\bar{\bar{x}}_m} + \phi_{\rm x}g^2\langle X_{\rm in}\rangle/\sqrt{2}, \quad \langle Y_{\rm out}\rangle = \tilde{\mu}_{Y_2|\bar{\bar{y}}_m} + \langle \tilde{Y}_1\rangle = \tilde{\mu}_{Y_2|\bar{\bar{y}}_m} + \phi_{\rm y}g^2\langle Y_{\rm in}\rangle/\sqrt{2}. \tag{19}$$

Accordingly, the quadrature variances of the teleported state can be expressed as

$$\begin{aligned}
\langle X_{\rm out}X_{\rm out}\rangle &= \langle \tilde{X}_2\tilde{X}_2\rangle + 2\phi_{\rm x}\langle \tilde{X}_2\tilde{X}_3\rangle + \phi_{\rm x}^2 g^2(\langle X_{\rm in}X_{\rm in}\rangle + C_{11})/2,\\
\langle Y_{\rm out}Y_{\rm out}\rangle &= \langle \tilde{Y}_2\tilde{Y}_2\rangle + 2\phi_{\rm y}\langle \tilde{Y}_2\tilde{Y}_1\rangle + \phi_{\rm y}^2 g^2(\langle Y_{\rm in}Y_{\rm in}\rangle + C_{22})/2.
\end{aligned} \tag{20}$$

Note that $\phi_{\rm x(y)}$ exploits the combined action of the classical rescaling factor $\beta = \lambda\sqrt{2}$ and the deterministic amplification with gain of ϵ addressed in the main text.

Fidelity and unity-gain condition. To examine the efficiency of our teleporter, we adopt the widely used criterion – fidelity – which quantifies the overlap between the input and the output states [4]. For Gaussian input states, the fidelity is given by

$$\mathcal{F} = \frac{2}{\sqrt{(\langle X_{\rm out}X_{\rm out}\rangle + \langle X_{\rm in}X_{\rm in}\rangle)(\langle Y_{\rm out}Y_{\rm out}\rangle + \langle Y_{\rm in}Y_{\rm in}\rangle)}}\exp\left[\frac{-1}{2}\left(\frac{(\langle X_{\rm out}\rangle - \langle X_{\rm in}\rangle)^2}{(\langle X_{\rm out}X_{\rm out}\rangle + \langle X_{\rm in}X_{\rm in}\rangle)} + \frac{(\langle Y_{\rm out}\rangle - \langle Y_{\rm in}\rangle)^2}{(\langle Y_{\rm out}Y_{\rm out}\rangle + \langle Y_{\rm in}Y_{\rm in}\rangle)}\right)\right]. \tag{21}$$

It is worthwhile noting that $\mathcal{F}$ becomes independent of the input amplitudes when the exponential term in (21) vanishes. This requires that $\langle X_{\rm out}\rangle = \langle X_{\rm in}\rangle$ and $\langle Y_{\rm out}\rangle = \langle Y_{\rm in}\rangle$, which is commonly refered to as the *unity-gain condition*. We emphasise that to construct a universal quantum teleporter capable of processing an arbitrary unknown input state, it is critical to fulfill the unity-gain condition to avoid any overestimation of the teleportation fidelity. In our scheme, the noiseless gains, denoted by g, and the rescaling operation, signified by $\phi_{\rm x(y)}$, complement each other to ensure the unity-gain condition is satisfied. As a result, the rescaling factors can be expressed as the function of g

$$\phi_{\rm x} = \frac{\sqrt{2}\left[\langle X_{\rm in}X_{\rm in}\rangle + C_{11} + C_{13}(g^2-1)\right]}{g^2(\langle X_{\rm in}X_{\rm in}\rangle + C_{11})} = \frac{\sqrt{2}\left[\langle X_{\rm in}X_{\rm in}\rangle + \cosh(2r) + (g^2-1)\sinh(2r)\right]}{g^2(\langle X_{\rm in}X_{\rm in}\rangle + \cosh(2r))}. \tag{22}$$

The attainability of the right hand side of Supplementary Equation (22) assumes that the original input squeezed states have similar squeezing levels of r which is satisfied in the our experimental demonstrations. Similarly, one can obtain $\phi_{\rm y}$, the rescaling applied onto $\tilde{Y}_1$, with $x \to y$ throughout. Note that the rescaling factor $\phi_{\rm x(y)}$ is independent of the input amplitude. When there is no noiseless amplification, namely $g = 1$, Supplementary Equation (22) reduces to $\sqrt{2}$, which coincides with the conventional teleportation [17]. When $g > 1$, $\phi_{\rm x(y)}$ is reduced to uphold the unity-gain condition.

Once the unity-gain condition is satisfied, the output quadrature variances can be re-arranged into a more succinct form by substituting Supplementary Equation (22) into Supplementary Equation (20)

$$\begin{aligned}\langle X_{\rm out}X_{\rm out}\rangle &= \frac{C_{11}-2C_{13}+C_{11}g^2+\langle X_{\rm in}X_{\rm in}\rangle-\frac{C_{13}^2(g^2-1)}{C_{11}+\langle X_{\rm in}X_{\rm in}\rangle}}{g^2},\\ \langle Y_{\rm out}Y_{\rm out}\rangle &= \frac{C_{22}+2C_{24}+C_{22}g^2+\langle Y_{\rm in}Y_{\rm in}\rangle-\frac{C_{13}^2(g^2-1)}{C_{11}+\langle Y_{\rm in}Y_{\rm in}\rangle}}{g^2}.\end{aligned} \tag{23}$$

In the context when the original squeezed states have the same squeezing levels r, the output variances in Supplementary Equation (23) can be re-arranged to yield the following formulas

$$\begin{aligned}\langle X_{\rm out}X_{\rm out}\rangle &= \frac{\langle X_{\rm in}X_{\rm in}\rangle\cosh(2r)+1}{\langle X_{\rm in}X_{\rm in}\rangle+\cosh(2r)}+\frac{(e^{-2r}+\langle X_{\rm in}X_{\rm in}\rangle)^2}{g^2\left(\langle X_{\rm in}X_{\rm in}\rangle+\cosh(2r)\right)},\\ \langle Y_{\rm out}Y_{\rm out}\rangle &= \frac{\langle Y_{\rm in}Y_{\rm in}\rangle\cosh(2r)+1}{\langle Y_{\rm in}Y_{\rm in}\rangle+\cosh(2r)}+\frac{(e^{-2r}+\langle Y_{\rm in}Y_{\rm in}\rangle)^2}{g^2\left(\langle Y_{\rm in}Y_{\rm in}\rangle+\cosh(2r)\right)}.\end{aligned} \tag{24}$$

For a sufficiently large noiseless gain g, the second terms in both $\langle X_{\rm out}X_{\rm out}\rangle$ and $\langle Y_{\rm out}Y_{\rm out}\rangle$ become negligible. The teleporter exhibits a very intriguing feature when operating in this regime: as long as the input state is pure, the output state remains pure, which means $\langle(\Delta X_{\rm out})^2\rangle\langle(\Delta Y_{\rm out})^2\rangle = 1$ regardless of the initial squeezing resource. This preservation of purity is only possible with infinite squeezing if a deterministic quantum teleporter is employed instead. For a pure input state that has $\langle(\Delta Y_{\rm in})^2\rangle = 1/\langle(\Delta X_{\rm in})^2\rangle$, the output purity of deterministic quantum teleportation is lower bounded by

$$\langle(\Delta X_{\rm out})^2\rangle\langle(\Delta Y_{\rm out})^2\rangle = \left(2e^{-2r}+\langle(\Delta X_{\rm in})^2\rangle\right)\left(2e^{-2r}+\frac{1}{\langle(\Delta X_{\rm in})^2\rangle}\right) \geq \left(2e^{-2r}+1\right)^2. \tag{25}$$

Only if $r\to\infty$, the teleported state is pure. Otherwise, an impure state is generated that is no longer a minimum-uncertainty state.

Consider an unknown coherent input state which sets $\langle(\Delta X_{\rm in})^2\rangle\langle(\Delta Y_{\rm in})^2\rangle = 1$, Supplementary Equation (23) can be significantly simplified given $r_x = r_y = r$.

$$\langle(\Delta X_{\rm out})^2\rangle = \langle(\Delta Y_{\rm out})^2\rangle = 1+\frac{2e^{-2r}}{g^2}. \tag{26}$$

The corresponding teleportation fidelity can be derived using Supplementary Equation (21) that yields

$$\mathcal{F} = \frac{1}{1+e^{-2r}/g^2}. \tag{27}$$

Supplementary Equation (27) signifies the enhancement in fidelity resulted from the noiseless linear amplification. In the limit of a sufficiently large g, the term e^{-2r}/g^2 tends to vanish irrespective of the initial EPR squeezing level. Therefore unit fidelity is always approachable without demanding more squeezing resource.

Supplementary Figure 2 (a) and (b) plot the teleportion fidelity as a function of the noiseless gain with different levels of squeezing resources. The input states in consideration here are a squeezed state of -3 dB and a coherent state, respectively. In both situations, significant improvement in fidelity is obtainable thanks to the noiseless linear amplification. In particular, with -6 dB of initial squeezing, our teleporter promises an output fidelity surpassing that is achievable using the conventional technique with currently best available squeezing of -15 dB [6]. These results significantly surmount the technical difficulties associated with the generation of high-magnitude squeezing. More remarkably, unit fidelity that would require infinite squeezing conventionally can be achieved with an arbitrary level of squeezing using the present scheme, provided that a sufficient noiseless gain is adopted.

Another interesting feature of our teleporter is its ability to purify thermal input states. In Supplementary Figure 3, we show the output variance as a function of the noiseless gain for teleporting a thermal state with variance of 3. In the absence of noiseless linear amplification denoting $g = 1$, additional noise penalty is enforced due to the finite level of the initial squeezing resource. The additional noise reduces as r increases; however, complete removal of the excess noise would ultimately require infinite squeezing, namely infinite energy. Our teleporter, on the contrary, offers a distinct advantage over its conventional counterpart. It not only circumvents the noise penalty, but also gives rise to a teleported state that has less noise than the original input state. The purification effect signifies one of the many unique properties of our teleporter. Intriguingly, less initial squeezing is in fact more beneficial for the sake of purification.

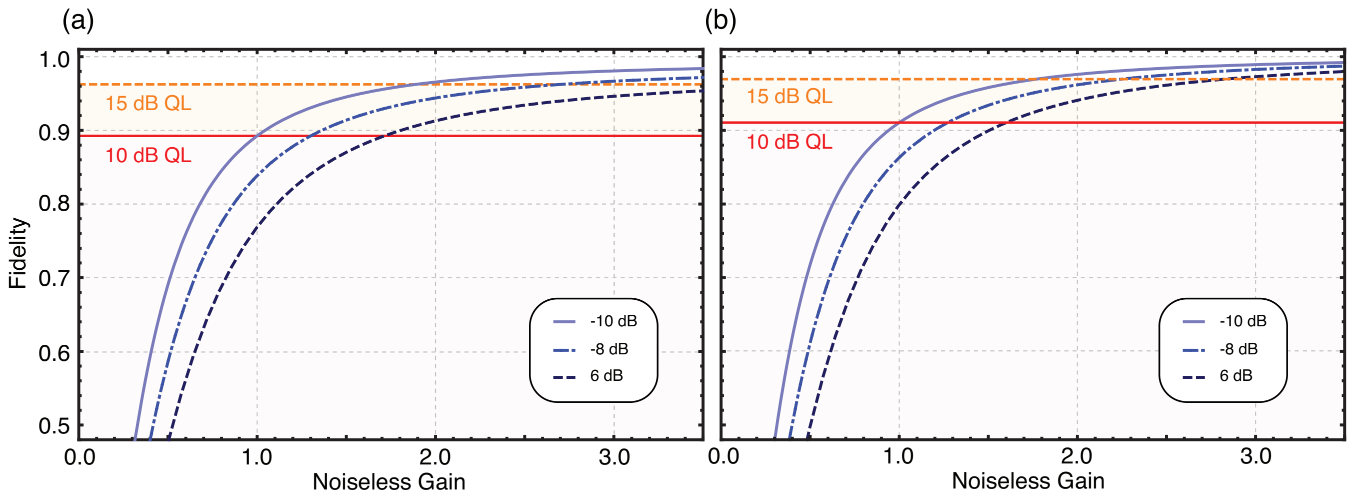

Supplementary Figure 2: Enhancement in fidelity as the noiseless gain increases for various initial squeezing levels: -6 dB (dashed), -8 dB (dash-dotted), and -10 dB. The input states to be teleported are a squeezed state of -3 dB (a) and a coherent state (b), respectively. For comparison, we plot the quantum limits when using initial squeezings of -10 dB and -15 dB , respectively. QL: quantum limit.

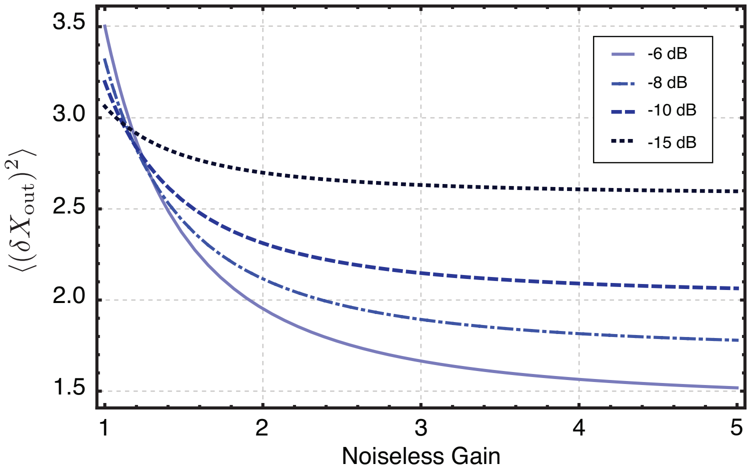

Supplementary Figure 3: Purification of a thermal input state with symmetric quadrature variance of 3 in phase and amplitude. The initial squeezing resource are -6 dB, -8 dB (dash-dotted), -10 dB (dashed), -15 dB (dotted), respectively.

Probability of success. Success probability can be derived from the probability distribution of the teleported state. For an input Gaussian distribution of $\alpha_{\rm m}$, the action of noiseless amplification is equivalent to multiplying $\alpha_{\rm m}$ by the filter function shown in Supplementary Equation (9). Upon proper normalisation of the output distribution, the success probability of the protocol can be cast in the following form [2].

$$P_{\rm s} = \frac{e^{(g-1)|\alpha_{\rm m}|^2}}{e^{\alpha_{\rm c}^2(1-1/g)}} \iint\limits_{|\alpha|<\alpha_{\rm c}} \exp\left(\frac{-|\alpha - g\alpha_{\rm m}|^2}{g}\right) {\rm d}^2\alpha + \iint\limits_{|\alpha|\geq\alpha_{\rm c}} \exp\left(-|\alpha-\alpha_{\rm m}|^2\right) {\rm d}^2\alpha \;. \tag{28}$$

Supplementary Figure 4 plots the probability of success as a function of the noiseless gain. It is worthwhile noting that the decrease of the success probability slows down as the noiseless gain increases, making our teleporter considerably practical for improving the efficacy of quantum teleportation.

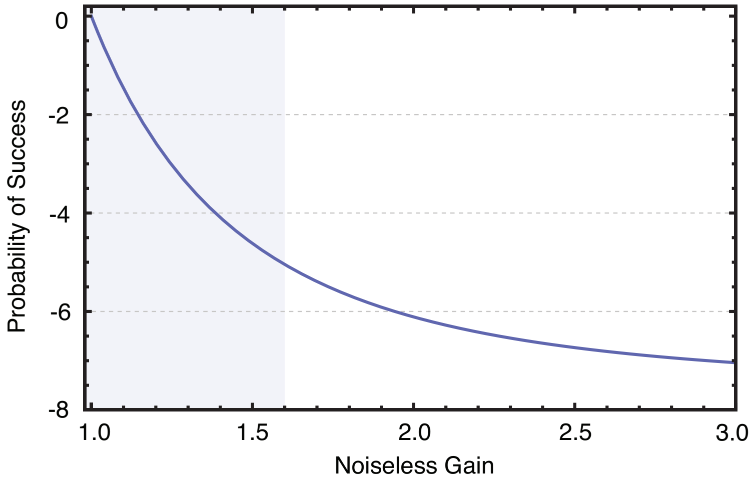

Supplementary Figure 4: Probability of success vs the noiseless gain in logarithmic scale. The shaded area indicates the experimental operation region in our demonstration. The cut-off parameter is $\alpha_c = 4.5$ which is chosen appropriately to avoid distortion of the output distribution [2].

II. SUPPLEMENTARY NOTE 2: NON-GAUSSIAN INPUT STATES

In this section, we provide a general framework to simulate our heralded quantum teleporter acting on arbitrary input states. The input state of the teleporter here is characterized by its Wigner function, denoted as $W_{\rm in}(\alpha_{\rm in})$, where the complex quadrature amplitude is $\alpha_{\rm mode} = (x_{\rm mode} + y_{\rm mode})/2$. Two single-mode input squeezed states, referred to as $W_{\rm sq_1}(\alpha_{\rm sq_1})$ and $W_{\rm sq_2}(\alpha_{\rm sq_2})$, are combined on a 50:50 beamsplitter to create a two-mode squeezed state, namely the EPR state. We assume equal levels of squeezing in the two modes. Recall the experimental schematic shown in Supplementary Figure 1, the combined mode comprised of the transmitted EPR (mode 2), and the two in-loop modes subject to the dual-homodyne station (mode 1 and 3) can be written as:

$$\begin{aligned}W(\alpha_2,\alpha_3,\alpha_1) = {} & W_{\rm sq_1}(\sqrt{t_{\frac{1}{2}}}\alpha_2 - \sqrt{(1-t_{\frac{1}{2}})t_{\frac{1}{2}}}\alpha_1 - \sqrt{(1-t_{\frac{1}{2}})(1-t_{\frac{1}{2}})}\alpha_3)\\ & \times\ W_{\rm sq_2}(\sqrt{(1-t_{\frac{1}{2}})}\alpha_2 + \sqrt{t_{\frac{1}{2}}t_{\frac{1}{2}}}\alpha_1 + \sqrt{t_{\frac{1}{2}}(1-t_{\frac{1}{2}})}\alpha_3)\\ & \times\ W_{\rm in}(\sqrt{t_{\frac{1}{2}}}\alpha_3 - \sqrt{(1-t_{\frac{1}{2}})}\alpha_1),\end{aligned} \tag{29}$$

where $t_{1/2}$ denotes the transmittivity of the beamsplitter being 50%. The subscripts $1,2,3$ denote the corresponding modes. Consider we measured the outcome (x_m, y_m) at the dual-homodyne, the probability of measuring the outcome is given by,

$$P_s(x_m,y_m) = \int_{-\infty}^{+\infty} d^2\alpha_2 dy_3 dx_1 W(\alpha_2, x_3 = x_m, y_3, x_1, y_1 = y_m). \tag{30}$$

The conditional transmitted mode can be expressed as

$$W_{\rm con}(\alpha_2|x_m,y_m) = \frac{1}{P_s(x_m,y_m)}\int_{-\infty}^{+\infty} dy_3 dx_1 W(\alpha_2, x_3 = x_m, y_3, x_1, y_1 = y_m). \tag{31}$$

The dual-homodyne outcomes are then selected with a weighting function $f(x_m, y_m)$, electronically rescaled by (ϕ_x, ϕ_y), and broadcast to Bob through a classical channel. Bob then performs a local displacement operation accordingly to reconstruct the input state. We obtain the output teleported state that takes the form,

$$W_{\rm out}(\alpha_2) = \frac{1}{P_{\rm out}}\int_{-\infty}^{+\infty} dy_m dx_m f(x_m,y_m) W_{\rm out}((x_2 + \phi_x x_m, y_2 + \phi_y y_m)|x_m,y_m). \tag{32}$$

Here $P_{\rm out}$ is the success probability of the heralded teleportation and is given by,

$$P_{\rm out} = \int_{-\infty}^{+\infty} d^2\alpha_2 dy_m dx_m f(x_m,y_m) W_{\rm out}((x_2 + \phi_x x_m, y_2 + \phi_y y_m)|x_m,y_m). \tag{33}$$

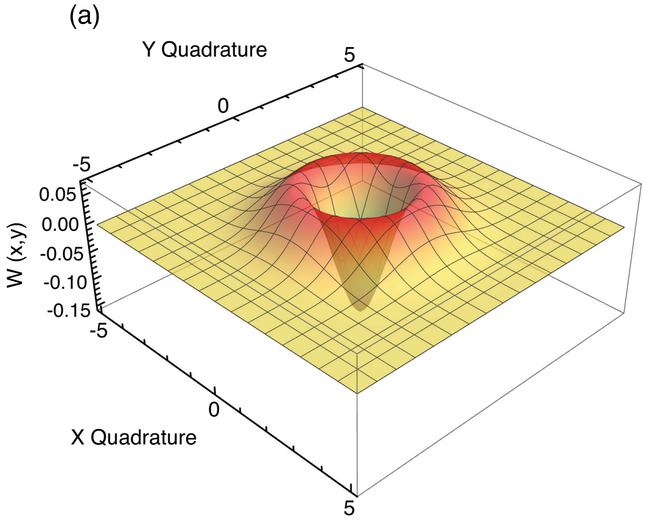

Supplementary Figure 5: **Output Wigner functions for non-Gaussian input states and the probability of success.** Given a -10 dB of initial squeezing, we obtain significant enhancement in fidelity for both a single-photon Fock input state and a cat state of amplitude 2.0. In the former case, we observe an output fidelity of 0.9977 as compared to $\mathcal{F}_{\rm DET} = 0.759$ if a conventional teleporter is used instead. In the latter case, our protocol gives a fidelity of $\mathcal{F} = 0.8729$ in contrast to $\mathcal{F}_{\rm DET} = 0.56$. The success probabilities in both cases are around 10^{-3} ($P_{\rm s} = 0.0087$ for the single photon input and $P_{\rm s} = 0.0030$ for the cat state input). The filter cut-off and noiseless gain used are 0.05 and 1.05, respectively.

The performance of the heralded teleporter is evaluated by measuring the overlap between the Wigner function of the teleported output state and the input state, which can be expressed as

$$\mathcal{F} = 4\pi \int_{-\infty}^{+\infty} d\alpha \, W_{\rm in}(\alpha) W_{\rm out}(\alpha). \tag{34}$$

We emphasize that the prime significance of the heralded teleporter is to show that the inclusion of NLA obviates the requirement of highly non-classical resources to achieve high performance quantum teleportation . The quantum resource would be necessary otherwise in conventional setup when benchmarked on the same criterion. In the above general diagram, the filter function $f(x_m, y_m)$ needs not to be restricted to $f(\alpha_m)$ as shown in Supplementary Equation (9). Instead, it can be tailored to optimise either the output fidelity, entanglement correlation, or to preserve maximally the input negativity, given *a priori* knowledge of the input alphabet. In the following, we explicitly consider two input non-Gaussian states, that is a single-photon Fock state and a Schrödinger's cat state with amplitude of 2.0. We show that by applying the following filter, we attain significant improvement in fidelity.

$$f(\alpha_{\rm m}) \propto \exp\left[\left(|\alpha_{\rm m}|^2 - \alpha_{\rm c}^2\right)(1 - \frac{1}{g^2})\right]. \tag{35}$$

The filter function closely resembles that in Supplementary Equation (9): similarly, measurement outcomes lying within the cut-off circle would be selected according to the acceptance rate $f(x_m, y_m)$; however, when the data ensemble falls beyond the cut-off, the points would be discarded instead of being kept with unity probability. Supplementary Figure 5 plots the Wigner functions of the teleported states. Considering -10 dB of initial squeezing, we obtain a near unit fidelity ($\mathcal{F} = 0.9977$) for a single-photon input Fock state in comparison to the fidelity of 0.759 if a conventional teleportation setup is used. For a cat input with amplitude of 2.0, the heralded teleporter gives a fidelity of 0.8729 as compared to 0.56 using the conventional setup, resulting in an improvement of $\sim$56%. We stress that the enhancement in fidelity comes at the expense of finite success probabilities. Although higher improvement is always achievable by increasing the noiseless gain, here we adopt a gain of 1.05 to maintain a practical success probability. In the context of teleporting non-Gaussian states, it is important that the negativity and quantum coherence of the input states are well preserved during the teleportation process. In Supplementary Figure 6, we present the 2D Wigner function in the phase quadrature of the input and output states. As evidenced in Supplementary Figure 6, we achieve significant improvement in retaining the negativity of the input states. In particular, for a single-photon input, the

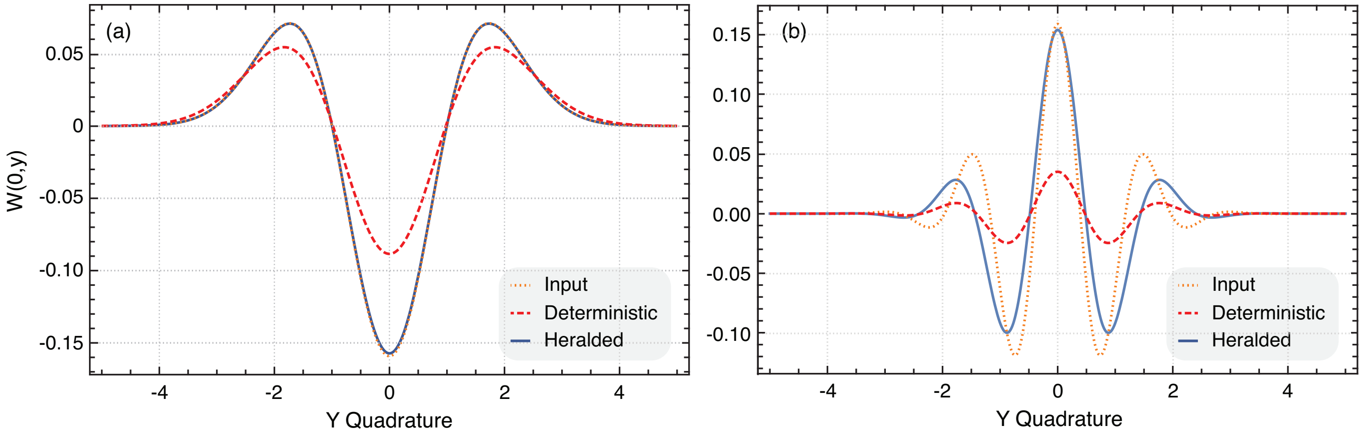

Supplementary Figure 6: **Wigner functions** $\mathrm{W}(0, y)$ **of the output states from the conventional and heralded quantum teleporters.** We assume -10 dB of squeezed resources in both cases. (a) shows $W(0, y)$ for an input Fock state and (b) for an input Schrödinger's cat state with amplitude of 2.0. In comparison with the conventional teleportation, our scheme offers apparent improvement in preserving the negativity of the Fock state and quantum coherence of the cat state. The filter cut-off α_c used is 0.05 and the noiseless gain is $g = 1.05$.

teleported state in the current scheme possesses almost identical negativity as the input, in stark contrast to the conventional scheme where the input negativity is largely impaired. The simulation assumes only a modest level of initial squeezed resource of -10 dB.

We note that conditional quantum teleportation relying on a bandpass filter, $x^2+y^2 \leq L^2$, has demonstrated to be useful for purifying a single-photon input state [7]. The negativity of the photonic state is resurrected by reducing the threshold parameter L. A higher purity is obtained by decreasing L, at the expense of a reduced success probability. The underpinning idea is to asymptomatically approach an ideal noiseless linear attenuator (NLAtt) signified by the transformation $|\alpha\rangle \rightarrow \exp\left[|\alpha|^2/2(1-g^2)\right]$. The exact noiseless attenuation is achieved when $L \rightarrow 0$, whereby the purity of the single photon state stays intact. One may notice that when operating our scheme with a noiseless gain $g < 1$ in Supplementary Equation (9), upon proper normalizations, our protocol effectively emulates a noiseless linear attenuator instead of a noiseless amplifier. In light of this discovery, we adopt a noiseless gain $g < 1$ for post-selection and analyze the action of our teleporter in this operation region on the same single photon and cat input states used above. We obtain output fidelity and success probability of $\mathcal{F} = 0.9976, P_s = 0.0085$ for the single photon input and $\mathcal{F} = 0.8732, P_s = 0.0034$ for the cat input state, respectively. The protocol works comparably to the scheme in [7], whereby the corresponding fidelity and success probability are $\mathcal{F} = 0.9976, P_s = 0.0087$ and $\mathcal{F} = 0.8732, P_s = 0.0035$, respectively. This is to be expected – the two protocols have similar performance in circumstances where the input states have probability distributions or quantum feature such as negativity centered at the origin.

However, note that the protocol in [7] essentially provides a measurement-based alternative to the physical implementations of a noiseless attenuator relying on single photon detectors [8–10]. Its operating region is consequently restricted to states with small amplitudes. In contrast, the operation regime of our protocol can in principle be arbitrarily large to encompass any input alphabet, at the expense of a decreasing success probability. To apply the protocol in [7] for input states containing a high number of photons, one can build an array of N such devices. The input state is evenly split among these N setups, with each constituent experiencing a noiseless attenuation. The N outputs of the NLAtt network are then combined to reconstruct the input state. The price to pay is that the success probability would decrease exponentially with the number of splitting. The scheme, in this scenario, resembles those proposed in [11, 12].

III. SUPPLEMENTARY NOTE 3: THEORETICAL MODE TAKING INTO ACCOUNT OF EXPERIMENTAL IMPERFECTIONS

In practice, the achievable fidelity depends on various empirical factors, such as the homodyne efficiency, dark noise clearance, and losses attributed to light propagation, impurity of the squeezing resource, and imperfect displacement operations. Here we provide a simple model of our teleporter by considering two dominant noise sources: excess in-loop dark noise, y_{DN} and x_{DN}, of the dual-homodyne detectors and the overall loss $\eta_{\mathrm{x(y)}}$ present in our setup on the amplitude (phase) quadrature.

The inclusion of dark noise and other loss results in a modified covariance matrix of the combined modes preceding post-selection. It can be derived by first re-arranging the covariance matrix in Supplementary Equation (4) to match the following

order of modes

$$(X_1, Y_1, X_2, Y_2, X_3, Y_3)^{\mathrm{T}} . \quad (36)$$

Second, two additional vacuum modes are coupled into the joint modes through virtual beamsplitters with transmittivities $\eta_{\mathrm{x}(y)}$. Non-unity transmission effectively emulate all downstream losses as light propagate through the system. Denote the covariance matrix in the new basis as Ω and a two-mode beamsplitter operation as $B(\eta)$, where η refers to the splitting ratio [4]. We obtain the covariance matrix prior to post-selection that includes the practical parameters:

$$\Omega' = \begin{pmatrix} B(\eta_{\mathrm{x}}) & 0 & 0 \\ 0 & I_2 & 0 \\ 0 & 0 & B(\eta_{\mathrm{y}}) \end{pmatrix} \begin{pmatrix} I_2 & 0 & 0 \\ 0 & \Omega & 0 \\ 0 & 0 & I_2 \end{pmatrix} \begin{pmatrix} B(\eta_{\mathrm{x}}) & 0 & 0 \\ 0 & I_2 & 0 \\ 0 & 0 & B(\eta_{\mathrm{y}}) \end{pmatrix}^T , \quad (37)$$

where I_{n} is an identity matrix with dimension of n. Effect of dark noise can be modeled by adding x_{DN} and y_{DN} to the quadrature noise $\langle X_3 X_3 \rangle$ and $\langle Y_4 Y_4 \rangle$, respectively. Substituting the corresponding covariance terms into Supplementary Equations (6) – (22) leads to the fidelity that takes into account of experimental imperfections. The practical loss parameters in our system are $\eta_{\mathrm{x}} \simeq 0.89$, $\eta_{\mathrm{y}} \simeq 0.9$, and $x_{\mathrm{DN}} = y_{\mathrm{DN}} = 0.01$.

IV. SUPPLEMENTARY NOTE 4: ENHANCEMENT IN THE DISTANCE OF TELEPORTATION

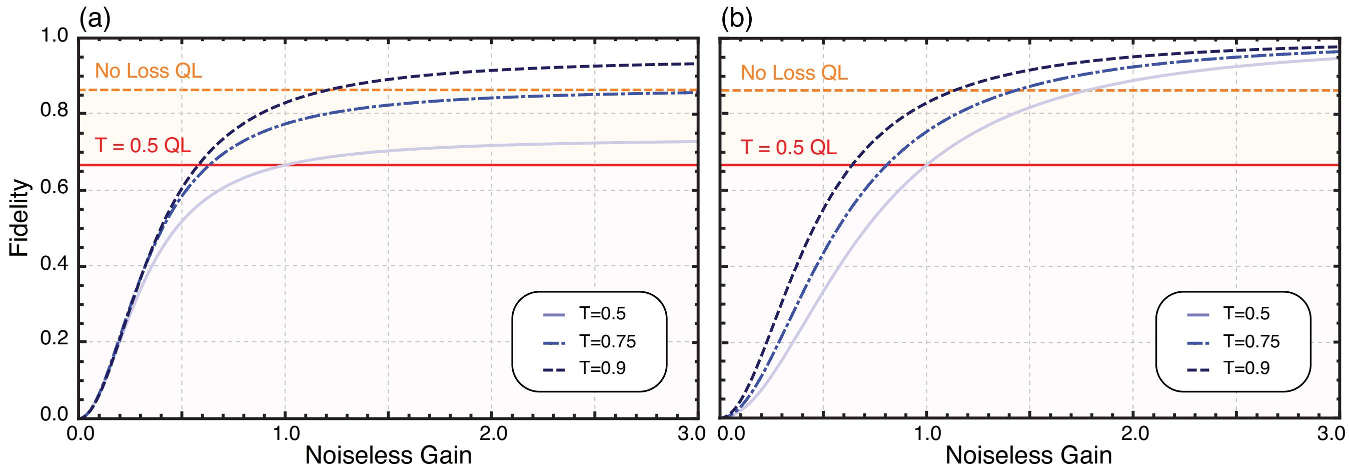

Supplementary Figure 7: Fidelity of teleportation when the distribution of entanglement is subject to imperfect channels. (a) illustrates the fidelity when different levels of loss, i.e. $T = 0.5$, $T = 0.75$, and $T = 0.9$, are imposed in Alices's channel, while (b) shows the fidelity in situations where Bob's EPR mode undergoes losses of $T = 0.5$, $T = 0.7$, and $T = 0.9$, respectively. The channel loss here is simulated by a partially transmissive beamsplitter. For comparison, the conventional deterministic teleportation when benchmarked by a pure channel (orange line) and a lossy channel with $T = 0.5$ (red dashed line) are superimposed. -8 dB of pure squeezed resource is assumed in all the plots.

As illustrated in Supplementary Figure 1, a quantum teleporter consists of two transmission channels: that from Victor to Alice and that from Victor to Bob. The overall teleportation distance is the sum of the two channels. In this section, we show how the present heralded quantum teleporter is capable of removing channel losses. This is of particular interest to achieve long-distance CV quantum teleportation. Unlike its discrete-variable counterpart that has realised teleporation of hundreds of killometers and ground-to-satellite teleportation [13–16], the distance of continuous-variable teleportation is substantially limited. This has been a long-standing issue in CV quantum information as teleportation serves as a key primitive for constructing a large-scale quantum network. The difficulty resides in the fact that the CV entanglement is extremely vulnerable to loss and noise. In practical implementations, long-distance distribution of entanglement would inevitably degrade the EPR correlation and hence the teleportation fidelity. Here we show that using our scheme, this technical hurdle can be nearly perfectly surmounted if the loss is imposed on Bob's EPR arm, and can be alleviated otherwise if the loss is injected on Alice's side. Theoretical model can be developed by simulating loss as a non-unity transmission coefficient, *i.e.* T through either Alice's or Bob's channel. For

example, in the former case, the covariance matrix of a lossy EPR pair is given by

$$\begin{pmatrix} B(T) & 0 \\ 0 & I_2 \end{pmatrix} \begin{pmatrix} I_2 & 0 \\ 0 & \mathbf{C} \end{pmatrix} \begin{pmatrix} B(T) & 0 \\ 0 & I_2 \end{pmatrix}^T, \tag{38}$$

where $\mathbf{C}$ refers to the lossless EPR modes shown in Supplementary Equation (1). The output quadrature means and variances, and hence the teleportation fidelity can be derived following the same methodology detailed in Supplementary Note I. Similarly, one can evaluate the performance of our teleporter when loss occurs in Bob's channel.

Supplementary Figure 7 shows the teleportation fidelity as a function of noiseless gain for various transmission coefficients. In Supplementary Figure 7 (a), we introduce loss on Alice's side, whereas in Supplementary Figure 7 (b) additional loss occurs on Bob's side. As shown in Supplementary Figure 7 (a), by increasing the noiseless gain, a higher fidelity can be achieved compared to the conventional teleporter–when benchmarked on the same channel loss (50%). More remarkably, for loss less than 25% (equivalent to 62 km of optical fiber) on Alice's side, we can obtain a fidelity beyond the best achievable via a conventional setup subject to a perfect channel. Further advantages of the present scheme lie in its absolute robustness against the loss on Bob's channel as plotted in Supplementary Figure 7 (b): regardless of the channel transmission, the noiseless amplification always allows us to compensate for the loss and hence obtain unit fidelity by turning up the noiseless gain although we note that our teleporter is heralded.

Comparison between Supplementary Figure 7 (a) and (b) showcases the asymmetric performance of our heralded teleporter, originating from the non-commutative nature of the beamsplitter operation and the noiseless linear amplification. An NLA executed before injecting the input state would result in a unit fidelity despite the asymmetry of the transmission channels. This no longer holds if the NLA is performed the other way around. The enhancement in fidelity in the present teleportation scheme is obtained by circumventing the noise penalty enforced on Alice's measurement on conjugate variables. As an infinite noiseless gain is applied, this additional noise can be completely avoided so the quadrature amplitudes of an unknown input may be inferred with arbitrarily high accuracy, and therefore, unit fidelity can be achieved.

V. SUPPLEMENTARY NOTE 5: TELEPORTING EPR STATES

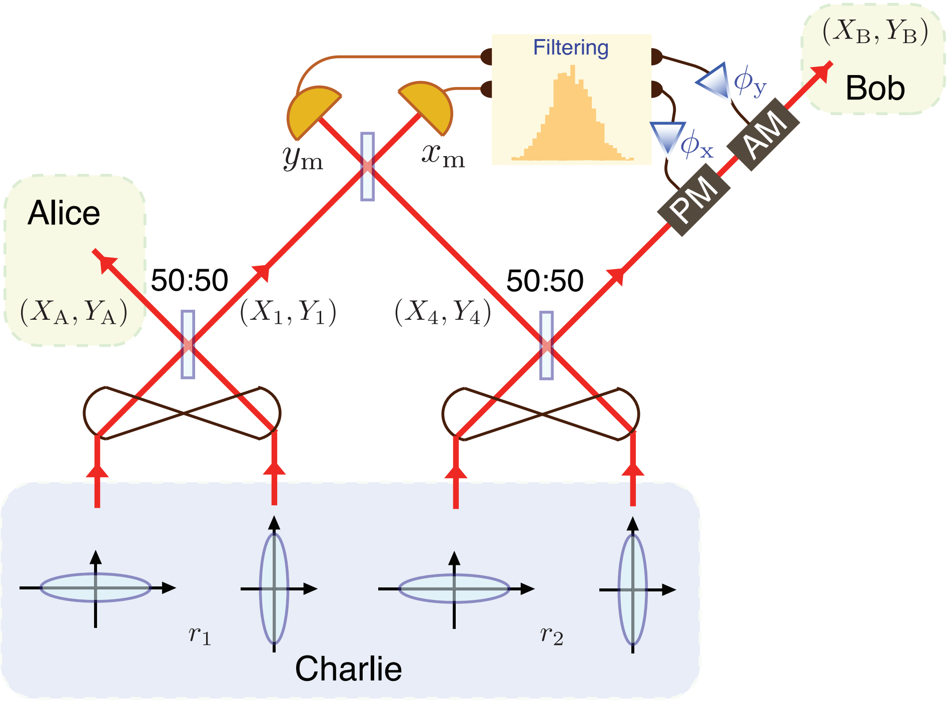

Supplementary Figure 8: Teleportation of one mode of an EPR pair. One arm of the input EPR state (squeezing parameter: r_1) is sent as the input state to our teleporter described in Supplementary Note I. Another EPR state with squeezing parameter r_2 is employed as the entanglement channel in teleportation. A filtering algorithm is embedded into the feed-forward loop to emulate a noiseless linear amplifier. With a successful heralding event, entanglement can be reliably distributed between Alice and Bob.

Teleportation of entanglement plays a pivotal role in long-distance quantum communication. Quantum signals during transmission inevitably experience loss and in some circumstances additional noise. This leads to the so-called decoherence phenomenon. Quantum decoherence deteriorates the non-classical features of the original signals, and therefore presents a major impediment to reliable quantum communication. One commonly known solution to this challenge is to build quantum repeaters to share entanglement between remotely located end users. Conventional quantum repeaters rely on entanglement distribution, entanglement swapping, and entanglement purification. In analogy to classical communication, the principle of quantum repeaters is to establish intermediate nodes so that quantum information can be relayed to end parties.

In the ensuing section, we study the performance of our heralded teleporter when used for teleporting EPR states. The scheme is illustrated in Supplementary Figure 8, which effectively constitutes an entanglement swapping protocol. The objective is to share entanglement between Alice and Bob who are far apart, so that Alice can directly teleport a quantum state to Bob using the entanglement channel established. We adopt the general framework developed in Supplementary Note II to investigate the performance of our heralded teleporter for teleporting EPR states. Note that this framework based on Wigner functions has also been used previously in analyzing deterministic swapping protocols [20]. We adopt the conventional figure of merit for entanglement swapping to evaluate the performance of our heralded teleporter in the context of teleporting EPR states, that is the purity of the output state and the output entanglement measured by entanglement of formation (EOF) which is an entanglement monotone [21]. We show that in this case, our teleporter outperforms the optimal conventional deterministic teleportation where one-sided displacements are employed [17–19].

We start with four single-mode squeezed states with squeezing parameters r_1, r_1, r_2 and r_2, as shown in Supplementary Figure 8. The respective covariance matrices are given by

$$\Sigma_{\mathrm{sq}_1}=\begin{pmatrix} e^{2r_1} & 0 \\ 0 & e^{-2r_1}\end{pmatrix}, \quad \Sigma_{\mathrm{sq}_2}=\begin{pmatrix} e^{-2r_1} & 0 \\ 0 & e^{2r_1}\end{pmatrix}, \quad \Sigma_{\mathrm{sq}_3}=\begin{pmatrix} e^{2r_2} & 0 \\ 0 & e^{-2r_2}\end{pmatrix} \quad \Sigma_{\mathrm{sq}_4}=\begin{pmatrix} e^{-2r_2} & 0 \\ 0 & e^{2r_2}\end{pmatrix}. \tag{39}$$

The Wigner function of the four-mode system is given by $W_{\mathrm{sq}_1}(\alpha_{\mathrm{sq}_1})W_{\mathrm{sq}_2}(\alpha_{\mathrm{sq}_2})W_{\mathrm{sq}_3}(\alpha_{\mathrm{sq}_3})W_{\mathrm{sq}_4}(\alpha_{\mathrm{sq}_4})$, where the complex amplitude is $\alpha_{\mathrm{mode}} = (x_{\mathrm{mode}} + iy_{\mathrm{mode}})/2$. The single-mode Wigner function $W_{\mathrm{sq}_\mathrm{m}}$ can be obtained from the corresponding covariance matrix $\Sigma_{\mathrm{sq}_\mathrm{m}}$ as follows

$$W_{\mathrm{sq}_\mathrm{m}}(\mathbf{x}) = \frac{\exp\left[-(1/2)(\mathbf{x}-\bar{\mathbf{x}})^T\Sigma^{-1}_{\mathrm{sq}_\mathrm{m}}(\mathbf{x}-\bar{\mathbf{x}})\right]}{2\pi\sqrt{\det\Sigma_{\mathrm{sq}_\mathrm{m}}}}, \tag{40}$$

where $\mathbf{x}$ is the vector of the quadrature variables, as defined in Supplementary Equation (5). The two squeezed beams with squeezing parameters r_2 are then combined on a 50:50 beamsplitter to form a two-mode squeezed state, also referred to as an EPR state, as the teleportation channel. Similarly, the remaining two squeezed states are combined to generate the EPR state to be teleported. The Wigner function of the combined state can be obtained by making the following substitutions.

$$\alpha_{\mathrm{sq}_1} \to \frac{1}{\sqrt{2}}(\alpha_2-\alpha_1), \quad \alpha_{\mathrm{sq}_2} \to \frac{1}{\sqrt{2}}(\alpha_2+\alpha_1), \quad \alpha_{\mathrm{sq}_3} \to \frac{1}{\sqrt{2}}(\alpha_4-\alpha_3), \quad \alpha_{\mathrm{sq}_4} \to \frac{1}{\sqrt{2}}(\alpha_4-\alpha_3). \tag{41}$$

The swapping is performed via the continuous-variable Bell measurement on modes 2 and 3 [17]. First, modes 2 and 3 are combined on a 50:50 beamsplitter, yielding outgoing modes α_u and α_v. This leads to the overall Wigner function prior to the measurement:

$$\begin{aligned} W_{\mathrm{BS}}(\alpha_1,\alpha_4,\alpha_\mathrm{u},\alpha_\mathrm{v}) = {} & W_{\mathrm{sq}_1}(-\frac{1}{\sqrt{2}}\alpha_1+\frac{1}{2}\alpha_\mathrm{u}-\frac{1}{2}\alpha_\mathrm{v})W_{\mathrm{sq}_2}(\frac{1}{\sqrt{2}}\alpha_1+\frac{1}{2}\alpha_\mathrm{u}-\frac{1}{2}\alpha_\mathrm{v}) \\ & \times W_{\mathrm{sq}_3}(\frac{1}{\sqrt{2}}\alpha_4-\frac{1}{2}\alpha_\mathrm{u}-\frac{1}{2}\alpha_\mathrm{v})W_{\mathrm{sq}_4}(\frac{1}{\sqrt{2}}\alpha_4+\frac{1}{2}\alpha_\mathrm{u}+\frac{1}{2}\alpha_\mathrm{v}). \end{aligned} \tag{42}$$

Then the quadratures x_u and y_v are measured using a dual-homodyne detection. Consider we measured the outcome $(x_\mathrm{m}, y_\mathrm{m})$ at the dual-homodyne, the Wigner function of mode 1 and 4 is obtained by integrating W_{BS} over the unmeasured quadrature variables y_u and x_v and setting $x_\mathrm{u} = x_\mathrm{m}$ and $y_\mathrm{v} = y_\mathrm{m}$:

$$W_{\mathrm{SIN}}(\alpha_1,\alpha_4 \mid x_\mathrm{u}=x_\mathrm{m}, y_\mathrm{v}=y_\mathrm{m}) = \frac{1}{P_\mathrm{s}(x_\mathrm{m},y_\mathrm{m})}\int\int dy_\mathrm{u}dx_\mathrm{v}W_{\mathrm{BS}}(\alpha_1,\alpha_4,x_\mathrm{u}=x_\mathrm{m},y_\mathrm{u},x_\mathrm{v},y_\mathrm{v}=y_\mathrm{m}). \tag{43}$$

Note that the normalization factor P_s denotes the probability (density) that the Bell measurement yields an outcome $(x_\mathrm{m}, y_\mathrm{m})$. In an idealized implementation, the probability to get an exact outcome $(x_\mathrm{m}, y_\mathrm{m})$ will be zero. The swapping scheme is referred to as *single-shot swapping* [20] where the conditional state W_{SIN} is an entangled state between mode 1 and 4 modulo a conditional displacement dependent upon the measurement outcome $(x_\mathrm{m}, y_\mathrm{m})$. Note that the conditional state W_{SIN} has covariance matrix

that is independent of the exact measurement outcome. The single-shot output $W_{\rm SIN}$, albeit its vanishing success probability, has the highest purity and entanglement and hence is the best achievable swapping result [20].

On the contrary, for deterministic EPR teleportation that is also referred to as *ensemble-average swapping*, the output is obtained by averaging the displaced conditional states $W_{\rm SIN}$ over all possible measurement outcomes weighted by the probability density for the respective outcome. Due to the randomness of the Bell measurement outcomes, the entangled output is subject to continuously fluctuating displacements [20]. These displacements can be undone partly by feedforwarding with optimal rescaling factors $\phi_{\rm x}, \phi_{\rm y}$ to mode 4. This conventional teleportation scheme constitutes the so-called *one-sided displacement swapping*. However, to fully undo the random displacements, one would need to feedforward with optimal rescaling factors to both modes 1 and 4, which is the optimal deterministic swapping protocol. It is worthwhile emphasizing that the optimal deterministic swapping is essentially a different scheme from the teleportation scheme. In the former case, the optimal operational regime is at gain-tuned and two-sided feedforward is required, whereas in the latter case, the teleporter should always operate at the unity-gain condition and only one-sided feedforward is necessary.

The feedforward enacts the following transformations of modes:

$$x_4 \to x_4' = x_4 + \phi_{\rm x} x_{\rm m}, \quad y_4 \to y_4' = y_4 + \phi_{\rm y} y_{\rm m}. \tag{44}$$

Accordingly, we obtain the overall output state of deterministic teleportation

$$W_{\rm DET}(\alpha_1, \alpha_4) = \iiiint dy_{\rm u} dx_{\rm v} dx_{\rm m} dy_{\rm m} W_{\rm BS}(\alpha_1, x_4 + \phi_{\rm x} x_{\rm m}, y_4 + \phi_{\rm y} y_{\rm m}, x_{\rm m}, y_{\rm u}, x_{\rm v}, y_{\rm m}). \tag{45}$$

The two-mode entangled state can in turn be used to teleport a coherent state under unity-gain condition. This way, we obtain the optimal rescaling factors for entanglement swapping by maximizing the teleportation fidelity [18, 20], which takes the form

$$\phi_{x(y)}^{\rm opt} = \mp \frac{\sqrt{2}(\sinh(2r_1) + \sinh(2r_2))}{\cosh(2r_1) + \cosh(2r_2)}. \tag{46}$$

In the following we analyze the performance of our heralded teleporter for EPR inputs. In contrast to the deterministic teleportation where the conditional states are averaged over all possible outcomes, the heralded quantum teleporter incorporates a weighting function $f(x_{\rm m}, y_{\rm m})$ on the measurement outcomes which takes the form of

$$f(\alpha_{\rm m}) \propto \exp\left[\left(|\alpha_{\rm m}|^2 - \alpha_{\rm c}^2\right)(1 - \frac{1}{g^2})\right]. \tag{47}$$

Similar to previous results where the heralded teleporter is employed to teleport coherent states, here the protocol has a finite success rate that depends on the post-selection gain g. Note that the weighting function takes a Gaussian form. In comparison to a notch-like filter that leads to similar performance, the Gaussian filter preserves the Gaussianity of the conditional states. The post-selection essentially modifies the probability density $P_{\rm s}(x_{\rm m}, y_{\rm m})$ of the Bell measurement outcomes based on which the conditional states are mixed up. As a result, the purity of the output can be improved. The overall output Wigner function can be expressed as

$$W_{\rm out}(\alpha_1, \alpha_4) = \frac{1}{P_{\rm out}} \iiiint dx_{\rm m} dy_{\rm m} dy_{\rm u} dx_{\rm v} f(x_{\rm m}, y_{\rm m}) W_{\rm BS}(\alpha_1, x_4 + \phi_{\rm x} x_{\rm m}, y_4 + \phi_{\rm y} y_{\rm m}, x_{\rm m}, y_{\rm u}, x_{\rm v}, y_{\rm m}), \tag{48}$$

where $P_{\rm out}$ is the success probability of the heralded teleportation that is given by

$$P_{\rm out} = \int d\alpha_1 d\alpha_4 dx_{\rm m} dy_{\rm m} dy_{\rm u} dx_{\rm v} f(x_{\rm m}, y_{\rm m}) W_{\rm BS}(\alpha_1, x_4 + \phi_{\rm x} x_{\rm m}, y_4 + \phi_{\rm y} y_{\rm m}, x_{\rm m}, y_{\rm u}, x_{\rm v}, y_{\rm m}). \tag{49}$$

The output Wigner function encompasses all the properties of the output state. For any two-mode Gaussian state with covariance matrix in the standard form

$$\Sigma = \begin{pmatrix} a & 0 & c_1 & 0 \\ 0 & a & 0 & c_2 \\ c_1 & 0 & b & 0 \\ 0 & c_2 & 0 & b \end{pmatrix}, \tag{50}$$

given its corresponding Wigner function W, the elements of the covariance matrix can be derived from the Wigner function as follows

$$\begin{aligned} a &= \iint d\alpha_1 d\alpha_4 W(\alpha_1, \alpha_4)(x_1 - \bar{x}_1)^2, \quad b = \iint d\alpha_1 d\alpha_4 W(\alpha_1, \alpha_4)(x_4 - \bar{x}_4)^2, \\ c_1 &= \iint d\alpha_1 d\alpha_4 W(\alpha_1, \alpha_4)(x_1 - \bar{x}_1)(x_4 - \bar{x}_4), \quad c_2 = \iint d\alpha_1 d\alpha_4 W(\alpha_1, \alpha_4)(y_1 - \bar{y}_1)(y_4 - \bar{y}_4), \end{aligned} \tag{51}$$

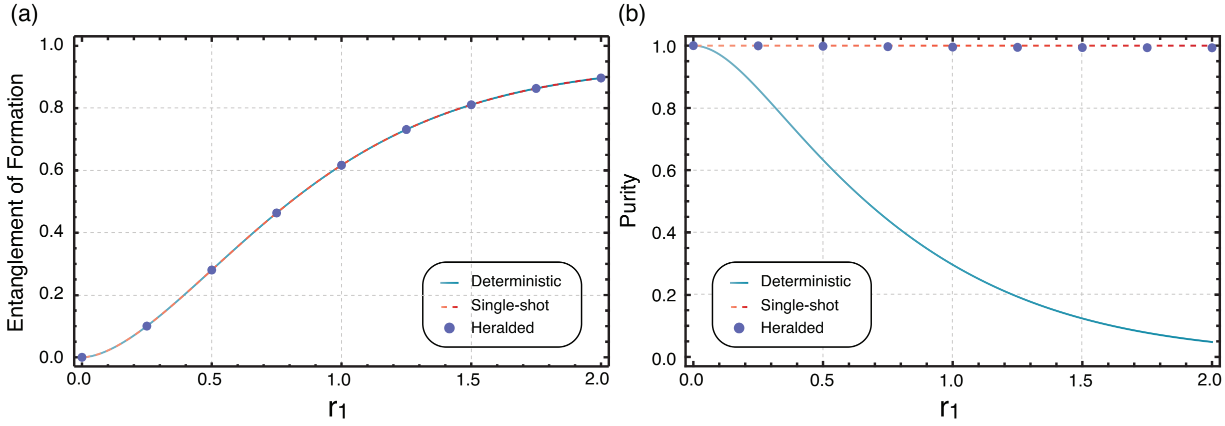

Supplementary Figure 9: Teleportation of one mode of an EPR pair in perfect transmission channel. (a) Entanglement of formation and (b) purity of the output two-mode states as a function of the input squeezing level r_1. The squeezing of the channel EPR r_2 is 0.5. The heralded teleporter yields two-mode states that have almost the same entanglement as the single-shot output (conditional state) and the deterministic swapping output. However, in contrast to the deterministic swapping where purity of the output states decreases as r_1 increases, the heralded teleporter yields output that has near-unit purity. The post-selection parameters are $\alpha_{\rm c} = 0.01$ and $g = 0.1$.

where $x(\bar{y})_{\rm n}$ are the respective quadrature mean amplitudes of mode n that can be obtained as follows

$$\bar{x}_{1(4)} = \iint d\alpha_1 d\alpha_4 W(\alpha_1, \alpha_4) x_{1(4)}, \quad \bar{y}_{1(4)} = \iint d\alpha_1 d\alpha_4 W(\alpha_1, \alpha_4) y_{1(4)}. \tag{52}$$

Supplementary Figure 9 compares the heralded teleporter to the optimal deterministic teleporter given EPR input states. Here we assume the channel EPR has a fixed squeezing level, namely $r_2 = 0.5$, whilst the squeezing of the input EPR r_1 is varied. The performance of the single-shot swapping is shown as an ultimate benchmark because both the purity and the EOF of any swapping protocol would be bounded from above by that of $W_{\rm SIN}$. As shown in Supplementary Figure 9 (a), the heralded and the deterministic teleportations have comparable performance in terms of the output entanglement. However, in contrast to the deterministic protocol which yields highly mixed states, the heralded teleporter effectively preserves the purity of the input state, regardless of the input squeezing level r_1. The enhancement in purity is more significant at higher r_1, at the expense of a reduced success probability (around $10^{-3} \sim 10^{-4}$ for all points). The fact that the deterministic teleportation scheme performs inferior to the single-shot swapping agrees with previous analysis [20] because one-sided displacement swapping protocol is not optimal.

Imperfect channel. In the following, we consider an imperfect transmission channel. Akin to [20], suppose the input EPR and the EPR resource have the same squeezing level, that is $r_1{=}r_2{=}r$, and the submode of the channel EPR that is sent over to the Bell measurement undergoes some loss characterized by a non-unity transmittivity T. The overall Wigner function prior to the Bell measurement can be derived accordingly:

$$\begin{aligned} W'_{\rm BS}(\alpha_1, \alpha_4, \alpha_{\rm u}, \alpha_{\rm v}) = {} & W_{\rm sq_1}(-\frac{1}{\sqrt{2}}\alpha_1 + \frac{1}{2}\alpha_{\rm u} - \frac{1}{2}\alpha_{\rm v}) W_{\rm sq_2}(\frac{1}{\sqrt{2}}\alpha_1 + \frac{1}{2}\alpha_{\rm u} - \frac{1}{2}\alpha_{\rm v}) \\ & \times W_{\rm sq_3}(\frac{1}{\sqrt{2}}\alpha_4 - \sqrt{\frac{1-T}{2}}\alpha'_3 - \frac{\sqrt{T}}{2}\alpha_{\rm u} - \frac{\sqrt{T}}{2}\alpha_{\rm v}) W_{\rm sq_4}(\frac{1}{\sqrt{2}}\alpha_4 + \sqrt{\frac{1-T}{2}}\alpha'_3 + \frac{\sqrt{T}}{2}\alpha_{\rm u} + \frac{\sqrt{T}}{2}\alpha_{\rm v}) \\ & \times W_{\rm vac}(\sqrt{T}\alpha'_3 - \sqrt{\frac{1-T}{2}}\alpha_{\rm u} - \sqrt{\frac{1-T}{2}}\alpha_{\rm v}). \end{aligned} \tag{53}$$

Here the transmission loss is characterized by a beamsplitter with transmittivity T and the mode α'_3 refers to the outgoing mode that is discarded. The respective single-mode Wigner functions can be obtained using Supplementary Equation (40). We consider pure lossy channel so that there is no excess noise. Following the same methodology, the conditional state, namely the single-shot swapping output, in this scenario can be derived by substituting $W_{\rm BS}$ in Supplementary Equation (43) by $W'_{\rm BS}$ in Supplementary Equation (53). Similary, the output of the deterministic teleportation can be obtained by making the substitutions

(Supplementary Equation (44)) that represents the action of the feedforward. The optimal rescaling factor in this case is given by

$$\phi_{x(y)}^{\mathrm{opt}'} = \mp \frac{\sqrt{2}(1+\sqrt{T})\mathrm{Sinh}(2r)}{1-T+(1+T)\mathrm{Cosh}(2r)}. \tag{54}$$

The results are summarized in Supplementary Figure 10. Same as before, the performance of the heralded and the deterministic teleportations are benchmarked by the single-shot swapping (red-dashed curve) that leads to the best output purity and entanglement of formation. Unlike in ideal transmissions, the heralded teleporter leads to significant enhancement in both entanglement of formation and the purity as compared to the deterministic teleportation. In comparison to the single-shot optimal swapping, the heralded teleporter yields output states with comparable purity and entanglement of formation; however, it is worth pointing out that our teleporter has a finite success probability ($10^{-3} \sim 10^{-4}$) whereas the single-shot swapping conditioned on an exact outcome at the Bell measurement would have vanishing success probability in an idealized implementation of the CV Bell measurement.

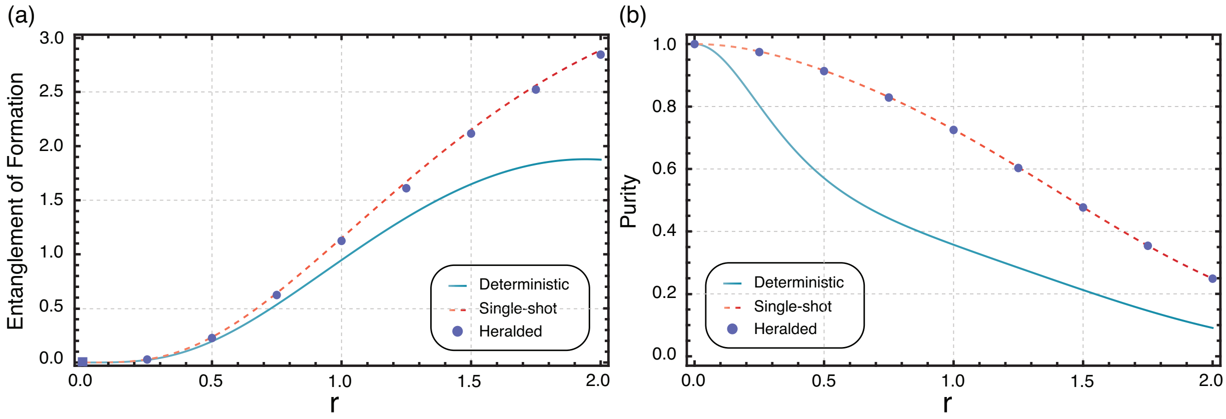

Supplementary Figure 10: Teleportation of one mode of an EPR pair in pure lossy channel. (a) Entanglement of formation and (b) purity of the output two-mode states as a function of r. Here both the input EPR squeezing level r_1 and the channel EPR squeezing level r_2 equal to r, that is $r_1 = r_2 = r$. The heralded teleporter achieves significant enhancement in entanglement as compared to the convention deterministic teleporter given optimal rescaling factors. As in the ideal channel, the heralded teleporter yields two-mode output states with comparable purity with the single-shot swapping; in contrast, the output of the deterministic teleportation is highly mixed, indicating its susceptibility to the negative effects of transmission losses. The post-selection parameters are $\alpha_c = 0.01$ and $g = 0.1$.

[1] Braunstein, S., L, & van Loock, P., Quantum information with continuous variables. Rev. Mod. Phys. 77, 513 (2005).

[2] Zhao, J., Hao, J. Y., Symul, T., Lam, P. K. & Assad, S. M. Characterization of a measurement-based noiseless linear amplifier and its applications. Phys. Rev. A 96, 012319 (2017).

[3] Blandino, R.. Walk, N., Lund, A., P., & Ralph, T., C., Channel purification via continuous-variable quantum teleportation with Gaussian postselection. Phys. Rev. A 93, 012326 (2016).

[4] Weedbrook, C., Pirandola, S., García-Patrón, R., Cerf, N. J., Ralph, T. C., Shapiro, J. H. & Lloyd, S. Gaussian quantum information. Rev. Mod. Phys. 84, 621 (2012).

[5] Braunstein, S. L. & Kimble, H. J. Teleportation of continuous quantum variables. Phys. Rev. Lett. 80, 869 (1998).

[6] Vahlbruch, H., Mehmet, M., Danzmann, K., & Schnabel, R. Detection of 15 dB squeezed states of light and their application for the absolute calibration of photoelectric quantum efficiency. Phys. Rev. Lett. 117, 110801 (2016).

[7] Fuwa, M., Toba, S., Takeda, S., Marek, P., Mista, L., Jr., Filip, R., van Look, P., Yoshikawa, J., & Furusawa, A. Noiseless conditional teleportation of a single photon. Phys. Rev. Lett. 113, 223602 (2014).

[8] Micuda, M., Straka, I., Mikova, M., Dusek, M., Cerf, N., J., Fiurasek, J., & Jezek, M. Noiseless loss suppression in quantum optical communication. Phys. Rev. Lett. 109, 180503 (2012).

[9] Ralph, T., C., & Lund, A., P. Nondeterministic noiseless linear amplification of quantum systems. AIP Conf. Proc. 1110, 155 (2009).

[10] Ulanov A. E., Fedorov I. A., Pushkina A. A., Kurochkin, Y. V., Ralph T. C.,& Lvovsky A. I. Undoing the effect of loss on quantum entanglement. Nat Photon. 9, 764 (2015).

[11] Ralph T. C., & Andersen U. L. High-fidelity teleportation of continuous-variable quantum states using delocalized single photons. Phys. Rev. Lett. 111, 0505054 (2013).
[12] Seshadreesan K. P., Krovi H., & Guha S. Continuous-variable quantum repeater based on quantum scissors and mode multiplexing. Phys. Rev. Research 2, 013310 (2020).
[13] Ma, X. S., Herbst, T., Scheidl, T., Wang, D., Kropatschek, S., Naylor, W., Wittmann, B., Mech, A., Kofler, J., Anisimova, E., Makarov, V., Jennewein, T., Ursin, R., & Zeilinger, A. Quantum teleportation over 143 kilometres using active feed-forward. Nature 269, 489 (2012).
[14] Valivarthi, R., Puigibert, M. G., Zhou, Q.,Aguilar, G. H., Verma, V. B., Marsili, F., Shaw, M. D., Nam, S. W., Oblak, D., & Tittel, W. Quantum teleportation across a metropolitan fibre network. Nature Photonics 10, 676 (2016).
[15] Ren, J. G., Xu, P., Yong, H. L., Zhang, L., Liao, S. K., Yin, J., Liu, W. Y., Cai, W. Q., Yang, M., Li, L., Yang, K. X., Han, X., Yao, Y. Q., Li, J., Wu, H. Y., Wan, S. W., Liu, L., Liu, D. Q., Kuang, Y. W., He, Z. P., Shang, P., Guo, C., Zheng, R. H., Tian, K., Zhu, Z. C., Liu, N. L., Lu, C. Y., Shu, R. S., Chen, Y. A., Peng, C. Z., Wang, J. Y., & Pan, J. W. Ground-to-satellite quantum teleportation. Nature 549, 70 (2017).
[16] Sun, Q. C., Mao, Y. L., Chen, S. J., Zhang, W., Jiang, Y. F., Zhang, Y. B., Zhang, W. J., Miki, S., Yamashita, T., Terai, H., Jiang, X., Chen, T. Y., You, L. X., Chen, X. F., Wang, Z., Fan, J. Y., Zhang, Q. & Pan, J. W. Quantum teleportation with independent sources and prior entanglement distribution over a network. Nature Photonics 10, 671 (2016).
[17] Braunstein S. L., & Kimble H. J., Teleportation of continuous quantum variables. Phys. Rev. Lett. 80, 869 (1998).
[18] van Loock, P., & Braunstein, S., L, Unconditional teleportation of continuous-variable entanglement. Phys. Rev. A 61, 010302 (R) (1999).
[19] Tan, S., M., Confirming entanglement in continuous variable quantum teleportation. Phys. Rev. A 60, 2752 (1999).
[20] Obermaier J. H., & van Loock P., Optimal Gaussian entanglement swapping. Phys. Rev. A 83, 012319 (2011).
[21] Tserkis S., & Ralph T. C., Quantifying entanglement in two-mode Gaussian states. Phys. Rev. A 96, 062338 (2017).
